# Decoding the Significance of Alpha Cell Function in the Pathophysiology of Type 1 Diabetes

**DOI:** 10.3390/cells13221914

**Published:** 2024-11-19

**Authors:** Jordan Carroll, Jessie Chen, Rahul Mittal, Joana R. N. Lemos, Mannat Mittal, Shreya Juneja, Amro Assayed, Khemraj Hirani

**Affiliations:** Diabetes Research Institute, University of Miami Miller School of Medicine, Miami, FL 33136, USA; jdc329@med.miami.edu (J.C.); jessiechen@med.miami.edu (J.C.); joanalemos@miami.edu (J.R.N.L.); 0528001@dadeschools.net (M.M.); juneja.shreya12@gmail.com (S.J.); assayedamr@gmail.com (A.A.)

**Keywords:** alpha cells, type 1 diabetes, glucose homeostasis, glucagon secretion, hypoglycemia, paracrine signaling

## Abstract

Alpha cells in the pancreas, traditionally known for their role in secreting glucagon to regulate blood glucose levels, are gaining recognition for their involvement in the pathophysiology of type 1 diabetes (T1D). In T1D, autoimmune destruction of beta cells results in insulin deficiency, which in turn may dysregulate alpha cell function, leading to elevated glucagon levels and impaired glucose homeostasis. This dysfunction is characterized by inappropriate glucagon secretion, augmenting the risk of life-threatening hypoglycemia. Moreover, insulin deficiency and autoimmunity alter alpha cell physiological responses, further exacerbating T1D pathophysiology. Recent studies suggest that alpha cells undergo transdifferentiation and interact with beta cells through mechanisms involving gamma-aminobutyric acid (GABA) signaling. Despite these advances, the exact pathways and interactions remain poorly understood and are often debated. Understanding the precise role of alpha cells in T1D is crucial, as it opens up avenues for developing new therapeutic strategies for T1D. Potential strategies include targeting alpha cells to normalize glucagon secretion, utilizing glucagon receptor antagonists, enhancing GABA signaling, and employing glucagon-like peptide-1 (GLP-1) receptor agonists. These approaches aim to improve glycemic control and reduce the risk of hypoglycemic events in individuals with T1D. This review provides an overview of alpha cell function in T1D, highlighting the emerging focus on alpha cell dysfunction in the context of historically well-developed beta cell research.

## 1. Introduction

Type 1 diabetes (T1D) is a chronic metabolic disorder characterized by the inability to produce insulin. The primary underlying pathophysiology of T1D is an autoimmune-mediated destruction of pancreatic β-cells, ultimately leading to insulin deficiency and a hyperglycemic state [1,2]. Although the primary pathological mechanism in T1D is the loss or dysfunction of β-cells, alpha cell dysfunction has also emerged as a burgeoning topic of investigation largely due to its unique role in glucose homeostasis considering the high burden of disease [3].

The latest epidemiological data reveals that of the 38.4 million Americans living with diabetes, 1.7 million (5.7%) have T1D, and that incidence is projected to increase in the coming decades [4,5]. Although once commonly referred to as juvenile diabetes, there has been an effort amongst the scientific community to phase out this term, as T1D can occur at any age, and this antiquated moniker may contribute to delayed or misdiagnosis of disease. As the population of the United States continues to grow, the prevalence of T1D in older adults is increasing, and they are the most at risk for disease-related complications and hypoglycemic events [5,6].

Management of T1D is aimed at controlling the plasma glucose concentration and keeping it within or as close to the normal blood glucose concentration of 70–180 mg/dL. By definition, T1D patients are insulin deficient and require exogenous insulin therapy [7]. Unfortunately, hypoglycemia is a common complication of insulin therapy and makes management of T1D challenging [7]. Another important component of management is minimizing time spent in both hypoglycemic and hyperglycemic states. Recently, the alpha cell, due to its unique role in glucagon secretion and increasing implication in the pathogenesis of T1D, has been gathering increased attention as a target for T1D treatment. The objective of this review is to provide a comprehensive overview of our current understanding of alpha cell physiology and pathology. We explore the role of alpha cells in the development of T1D and their potential as a target for treatment.

## 2. Importance of Alpha Cell Function in Glucose Homeostasis

Glucose homeostasis is maintained by the concerted and opposing actions of insulin and glucagon, which have been described as functional antagonists [8]. Alpha cells of the human pancreas are primarily responsible for the production and secretion of glucagon, making them integral to maintaining plasma glucose concentrations within normal physiological ranges [9,10,11,12,13,14]. Studies in both isolated islets and purified alpha cells have demonstrated that glucagon is solely secreted by alpha cells in response to insulin inhibition or somatostatin signaling [15,16]. In response to hypoglycemia, healthy alpha cells are stimulated to release glucagon. Glucagon counteracts insulin-driven hypoglycemia by stimulating both glycogenolysis and gluconeogenesis in the liver, raising plasma glucose levels [17,18]. Glycogenolysis and gluconeogenesis primarily occur in the liver and, to a lesser extent, in the kidneys [19,20]. These processes are tightly regulated by glucagon secreted from pancreatic alpha cells, which stimulates hepatic glycogen breakdown and glucose production to maintain blood glucose levels, especially during fasting states [21,22]. In the liver, glucagon promotes both glycogenolysis and gluconeogenesis, while in the kidneys, it mainly supports gluconeogenesis under prolonged fasting or stress conditions. This interplay between alpha-cell glucagon release and glucose metabolism in peripheral organs highlights the systemic coordination needed for glucose homeostasis. 

Functioning alpha cells are critical in maintaining glucose homeostasis, as patients with diabetes lose the ability to appropriately regulate their blood glucose levels. Alpha call dysfunction and the complications consequently experienced by T1D patients are two-pronged. Patients experience both aberrantly elevated glucagon levels (hyperglucagonemia), further exacerbating their hyperglycemia, and impaired alpha cell response to iatrogenic hypoglycemia secondary to insulin therapy [17]. 

## 3. Basic Anatomy and Physiology of Alpha Cells 

### 3.1. Location and Structure of Alpha Cells 

**Figure 1:**
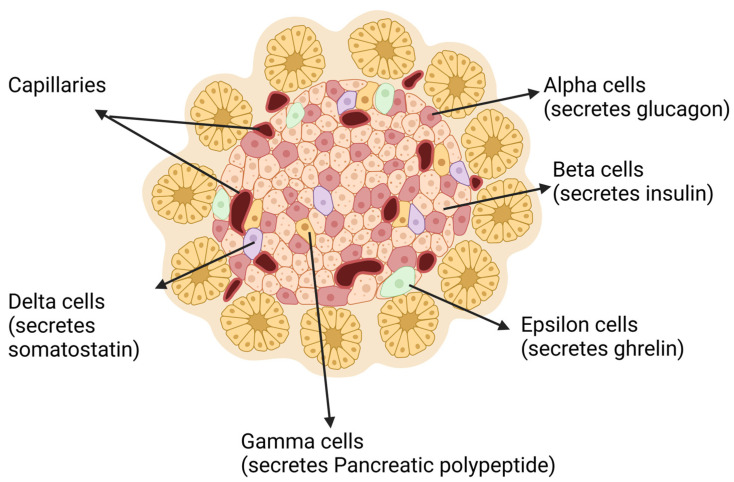
A schematic representation of various cell types within the pancreatic islet. Alpha cells secrete glucagon, whereas beta cells, the most abundant cell type, are dispersed throughout the islet and produce insulin. Delta cells, interspersed among alpha and beta cells, secrete somatostatin, which regulates the function of nearby cells. Additionally, gamma cells (also named as PP cells), found sparsely in the islet, produce pancreatic polypeptide. Epsilon cells produce ghrelin. “Created in BioRender. Mittal, R. (2024). https://BioRender.com/r09m051 (accessed on 1 September 2024)”.

Alpha cells are one of the key components of healthy pancreatic islets of Langerhans, which have been elegantly described as endocrine micro-organs [23]. Each pancreatic islet is a heterogeneous mix of alpha, beta, delta, epsilon, and gamma (also named PP cells) endocrine cells, with each islet having a unique arrangement and irregular structure [24] (Figure 1). Alpha cells, second only to beta cells, constitute 20–40% of pancreatic mass, which is preserved under normal conditions of aging despite loss of exocrine components [25,26]. Despite geographic variation in alpha cell location within individual islets, most alpha cells in healthy rodent tissue are found in the periphery of each islet and in close proximity to beta cells [27]. This distinct arrangement is a characteristic feature observed in murine models and plays a role in how islet cells interact and regulate hormone secretion [28]. In contrast, human islets exhibit a more dispersed organization, with alpha cells intermingled among beta cells throughout the islet rather than restricted to the periphery. This more homogeneous distribution allows for closer intercellular communication within human islets, which could impact the regulatory mechanisms of glucagon and insulin secretion [29,30,31]. Understanding these structural differences is crucial for interpreting studies on islet cell function and for translating findings from rodent models to human biology.

### 3.2. Alpha Cell Secretions and Their Mechanisms 

**Figure 2:**
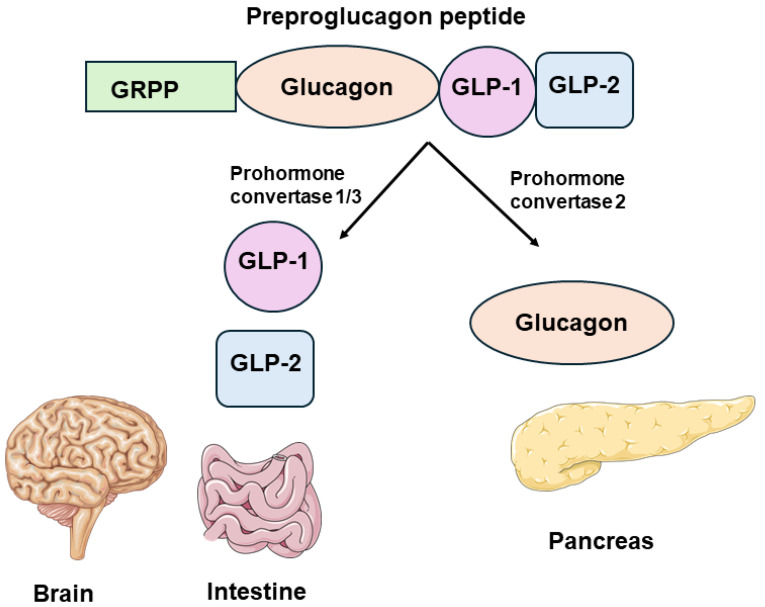
A schematic representation of post-translational processing of the preproglucagon peptide: In the pancreas, PC2 processes the proglucagon peptide to produce glucagon. In the gut and nervous system, PC1/3 processes it to generate GLP-1 and GLP-2. Some of the images were taken from Servier Medical Art templates, which are licensed under a Creative Commons Attribution 4.0 License. https://smart.servier.com (accessed on 1 September 2024).

Glucagon is the main secretory product of alpha cells and is the result of a post-translational modification of its precursor preproglucagon, encoded by the *GCG* gene. Pancreatic alpha cells express prohormone convertase 2 (PC2), which cleaves preproglucagon into glucagon [11,14,18]. A schematic representation of post-translational processing of the preproglucagon peptide in the pancreas and other organs such as the gut and nervous systems has been shown in Figure 2. This tissue-specific enzymatic processing is conserved across species, supporting similar roles for glucagon and glucagon-like peptides (GLP) in both rodents and humans [32,33,34]. Once synthesized, glucagon is stored in large, dense-core secretory granules within alpha cells.

Release of glucagon from alpha cells is a calcium-dependent process. In response to depolarization of the cell membrane, glucagon secretory granules fuse with the plasma membrane, and rapid exocytosis of the contents ensues. A study of pancreatic islet cell architecture using scanning electron microscopy (SEM) found that alpha cells have more extensive endoplasmic reticulum systems than beta cells, which contribute to enhanced calcium-induced calcium release (CICR) [35,36]. This enhanced CICR, along with more densely packed glucagon granules and smaller cell diameter, is thought to enable rapid, high-volume glucagon release [35,36]. Further investigation is warranted to determine if these characteristics are present in human alpha cells.

Another key feature of pancreatic alpha cells is the presence of melatonin and its receptors, primarily MT1 and MT2 [37,38], though these receptors are expressed at lower densities in alpha cells [39]. These receptors influence the release of pancreatic hormones from alpha, beta, and delta cells [40,41]. However, there is contradictory evidence regarding receptor expression in alpha cells. Some studies suggest that both MT1 and MT2 are expressed at similarly low levels, while others report very low or absent MT2 expression, indicating that MT1 may be the dominant receptor involved in melatonin-regulated glucagon secretion [42,43]. Another study further demonstrated in vitro and in vivo that melatonin stimulates glucagon secretion, with this effect being exacerbated under hyperglycemic conditions [44]. Studies in T1D patients identified elevated plasma melatonin levels and an inverse relationship with insulin, aligning with findings in rodent models [45]. Further studies are needed to fully characterize the changes in melatonin receptor density in T1D.

### 3.3. Interactions with Beta Cells

In response to hyperglycemia, beta cells of the pancreas synthesize and secrete the hormone insulin to lower serum glucose levels. In healthy individuals, the glucose-lowering action of beta cells is opposed by alpha cells and their secreted glucagon product [46]. The link between alpha and beta cells in maintaining glucose homeostasis is evident; however, the exact mechanisms by which these cells communicate are still highly debated and investigated. It has been hypothesized that alpha and beta cells communicate via paracrine signaling and that optimal insulin secretion is dependent on these paracellular interactions [47,48]. The relative proximity of these cell types to each other, along with the heterogenic organization of pancreatic islets, has been used to support the dominant model of paracrine signaling in alpha and beta cell physiology.

Historical pancreatic perfusion studies have long suggested the existence of a “core-mantle” vascular arrangement in pancreatic islets. In this proposed arrangement, blood flows from beta cell-rich islet cores to predominantly alpha cell-rich islet mantles, with circulation independent of the exocrine pancreas. Under this vascular model, insulin secreted by beta cells is thought to flow downstream and suppress glucagon secretion from alpha cells [49]. Importantly, recent evidence from dynamic 3D image islet modeling and in vivo studies cast doubt that this core-mantle model, developed from 2D images, adequately characterizes islet vasculature and blood flow in vivo. Dybala et al. instead describe islet blood flow as an “open circulation” with extensive communication between the endocrine and exocrine pancreas, where blood flow and thus cell secretory products flow bidirectionally to exert their effects [50].

It is important to note that experimental methods that have been historically used to investigate pancreatic islet cell interactions disrupt the normal architecture and thus the paracrine signaling of alpha and beta cells. Static perfusion and perfusion of isolated islets, although useful, disrupt the normal environment, architecture, and vascularity of pancreatic islets, which can affect experimental outcomes and call into question the reliability and relevance of results obtained using these methods. 

## 4. Pathophysiology of Alpha Cells in Type 1 Diabetes 

### 4.1. Alpha Cell Dysfunction in Type 1 Diabetes

The current beta cell-dominant framework for the discussion of the pathophysiology of T1D fails to emphasize the important role that alpha cell dysfunction contributes to the development of T1D. In contrast to beta cell mass, alpha cell mass is preserved in T1D patients early in disease progression [12]. Despite initial mass preservation, which may occur due to both autoimmune destruction of beta cells and enhanced alpha cell proliferation from chronic hyperglycemia, T1D patients exhibit profound alpha cell dysfunction, aberrant gene expression, and altered proliferation as the disease progresses [12]. Importantly, this dysfunction is believed to begin prior to the destruction of beta cells. Furthermore, cytokine-regulated genes and genes of glucagon biosynthesis have been found to be upregulated, as have endoplasmic reticulum stress genes in alpha cells [51]. Ultimately, there is a reduction in both beta and alpha cell mass in T1D that contributes to further disease progression [52,53]. With these recent discoveries, more emphasis and research have been directed toward strategies that target alpha cells to treat T1D.

### 4.2. Impact of Insulin Deficiency on Alpha Cells

Administration of exogenous insulin in T1D therapy exacerbates the dysregulation of alpha cells and, in cases where they are not able to mount an appropriate response, may cause life-threatening hypoglycemia. This is due to the chronically elevated level of insulin from the supplemental therapy that prevents alpha cells from detecting low beta cell stimulation [54]. Furthermore, there is growing evidence that pancreatic endocrine cell lineages are impacted by insulin function [55].

### 4.3. Role of Autoimmunity in Alpha Cell Alteration

A perplexing feature of the auto-immune-driven changes seen in the progression of T1D is that despite dysregulation occurring in both alpha and beta cells, alpha cells survive while beta cells are destroyed. A recent perspective has highlighted this perplexing feature of autoimmune alteration in alpha cells and suggested that differential gene expression, including enhanced expression of BCL21 (anti-apoptotic), differing expression of key endoplasmic reticulum stress-related genes, and higher expression of viral recognition and innate immune response genes, confer a survival advantage to alpha cells despite their maintained dysregulation [52]. 

## 5. Alpha Cell’s Role in Glucose Regulation 

### 5.1. Normal Function of Alpha Cells in Glucose Homeostasis

The differentiation of alpha cells is mediated by various genes: mainly *Pdx1* (pancreatic and duodenal homeobox 1) and *Ngn3* (neurogenin-3), as well as others such as *Arx* (aristaless-related homeobox), *Fox-A2* (forkhead box protein A2), and *Pax4* (paired box 4) [56]. A recent study has implicated ISL1 as a primary controller of alpha cell fate and a major regulator of beta cell maturation [57]. 

Alpha cells have various types of ion channels, such as voltage-gated (P/Q-type, L-type, and N-type) calcium channels, ATP-sensitive potassium channels, and voltage-gated sodium channels that can help generate action potentials, but this is highly dependent on the extracellular glucose concentration [58]. The proposed model by which this mechanism occurs is the “regenerative model”. When there is a drop in extracellular glucose concentration, as in hypoglycemia, the ATP/ADP ratio drops, which activates ATP-sensitive potassium channels and voltage-gated sodium channels to raise the membrane potential to around −60 mV [56,58,59]. This increase in membrane potential is enough to activate T-type calcium channels, further depolarizing the membrane to activate voltage-gated sodium channels and L-type, P/Q-type, and N-type calcium channels. This large intracellular influx of calcium is crucial for glucagon vesicle exocytosis and in turn for glucagon secretion. The repolarization of the cell is then regenerated by voltage-gated potassium channels [56].

However, a separate model suggests that high glucose inhibits secretion of glucagon independently of ATP-sensitive potassium channels, and instead, there is suppression of calcium store-operated current depolarization [58]. It has been demonstrated that hypoglycemic conditions led to low ATP-sensitive potassium channel activity and hyperglycemic conditions led to complete inhibition of the channel’s activity [58]. The current controversy warrants further study into the exact mechanism of glucagon release from alpha cells. Overall, based on studies conducted within in vitro human islet cells and mouse models, ATP plays a key role, and the balance between fatty acid oxidation, and glucose metabolism mediates this process in alpha cells, mostly due to fatty acid oxidation the amount of intracellular ATP available, and therefore inhibiting glucagon secretion [60].

Once glucagon is secreted, it is engaged in autocrine feedback leading to the release of more glucagon in hypoglycemic states [56]. Furthermore, glucagon-like peptide (GLP-1) and glucose-dependent insulinotropic polypeptide (GIP) are incretins that can exert autocrine activity as well to respectively reduce glucagon secretion, and thus they also play a role in glucose homeostasis [58,61]. GLP-1 exhibits hypoglycemic activity that works in conjunction with glucose-dependent insulin secretion, inhibition of glucagon production, and regulation of islet cell life course [62]. GIP has also been demonstrated to play a role in enhancing the trans-differentiation of alpha cells to beta cells as T1D progresses in streptozotocin mice, which may serve as a therapeutic approach for glycemic control that warrants further modeling and investigation [63]. Currently, there is also potential in creating chimeric peptides of GIP and GLP-1 to modulate glucose control and weight loss in obese diabetic individuals [64].

Conversely, in high extracellular glucose concentration, beta cells normally inhibit glucagon secretion (such as in a postprandial state) [56]. It has been reported that alpha cells have a myriad of hormone receptors that can have inhibitory or stimulatory effects [58]. The major inhibitory hormone of alpha cells is insulin, as in high extracellular glucose states [58]. Furthermore, somatostatin also exerts an inhibitory effect on alpha cells and prevents glucagon release [58,59]. Contrastingly, glucagon and acetylcholine secretion in hyperglycemic states can enhance insulin secretion to counteract the increased glucose levels [56]. Interestingly, vitamin D-binding protein (DBP), which binds vitamin D metabolites, has recently been discovered to play a role in alpha cell function, as deletions in DBP have implications for glucagon secretion, response to hypoglycemia, and alpha cell mass, which may all play a role in the pathophysiology of T1D [65].

### 5.2. Dysregulation of Glucagon Secretion in Type 1 Diabetes

While alpha cell involvement in T1D has not been fully characterized, Unger and colleagues present evidence that glucagon hypersecretion plays a critical role in T1D hyperglycemia [58]. Disrupting glucagon receptor signaling and utilizing glucagon receptor antagonists to shed light on the role of glucagon points to evidence that glucagon plays a role in T1D. When rodents and humans with diabetes were treated with glucagon receptor antagonists, the results demonstrated improved glycemic control [58].

One recently proposed hypothesis regarding the mechanism of the abnormal glucagon secretion in T1D is the loss of intra-islet capillaries and nerve fibers secondary to a reduction in beta cell mass. Comparing non-diabetic, recent-onset (<10 years), and longstanding (>10 years) T1D donor pancreatic tissues, recent-onset T1D tissues demonstrated more islet and acinar capillary density than non-diabetic and longstanding T1D, and recent-onset and longstanding T1D tissues had more nerve fiber density than non-diabetic tissues [66]. The persistence of the nerves and capillaries suggests that the abnormal glucagon secretion is independent of the vascular and neural components. Currently, there is evidence that this abnormal glucagon secretion has implications in exocrine pancreatic atrophy; however, further investigation is warranted to elucidate this mechanism [67]. In addition, the destruction of insulin-mediated beta cell inhibition of alpha cells contributes to alpha cell hypertrophy and the eventual development of hyperglucagonemia and hyperglycemia [58].

## 6. Clinical Implications of Alpha Cell Dysfunction 

### 6.1. Hypoglycemia in Type 1 Diabetes

As discussed previously, individuals with diabetes are more susceptible to hypoglycemia as they lose the ability to effectively regulate their plasma glucose levels [9,68]. Patients with more severe T1D require more time for glucose levels to normalize after insulin-induced hypoglycemia and experience prolonged low plasma glucagon levels, highlighting the role of glucagon in managing hypoglycemia [3]. Insufficient glucagon secretion during hypoglycemic episodes raises the risk of hypoglycemic shock, while excessive glucagon in a postprandial state can worsen hyperglycemia [69]. Taken together, these points illustrate that while glucagon is necessary to prevent hypoglycemia, it also has diabetogenic effects [9]. Further research is needed to better understand glucagon’s role in alpha cell dysfunction in diabetes.

Furthermore, hypoglycemia is associated with high expression of the receptor for advanced glycation end products (RAGE) in T1D adolescent donors compared to non-diabetic adolescent donors, with *AGER* being the most important predictor of the islet’s glucagon levels [70]. While the correlation between this receptor and glucagon has been established, further investigation into the role of RAGE in T1D should be conducted, especially in the context of T1D. 

### 6.2. Complications Arising from Alpha Cell Dysfunction

Increased alpha cell glucagon secretion in diabetes and lack of response to glucose levels has been attributed to the lack of inhibition of cAMP production by serotonin produced from beta cells, as cAMP increases baseline levels of glucagon secretion [9]. In diabetes, this leads to a new baseline glucagon level that is elevated. Furthermore, this has been characterized as independent from changes in insulin secretion from beta cells; one experiment performed on isolated islets from single autoantibody-positive donors demonstrated that human alpha cells have altered expression of important transcription factors that affect the expression of genes in glucose metabolism, endoplasmic reticulum, electrical activity, and calcium signaling in T1D [69,71]. In a separate experiment conducted on Type 2 Diabetes Mellitus (T2D) human donor alpha cells, the results demonstrated that glucagon exocytosis is not prevented by hyperglycemia or insulin inhibition [56]. Moreover, deleting insulin receptor-coding genes led to hyperglucagonemia and hyperglycemia [56]. T1D individuals tend to have hyperglucagonemia despite hyperglycemic states due to alpha cell dysfunction [58,72]. There is evidence pointing towards alpha cell hypertrophy and hyperplasia and the loss of beta cell inhibition through insulin [58].

Hyperglucagonemia and alpha cell proliferation have been reported in subjects with T1D. For example, in a streptozotocin mouse model, the administration of a positive allosteric modulator in conjunction with the activation of the sirtuin pathway improved hyperglycemia and hyperglucagonemia and decreased alpha cell proliferation. However, in another study, findings in T1D adolescents and young adults suggest that alpha cell proliferation and hypertrophy may not be contributing significantly to the clinical implications [58]. Further investigation into the relationship between these factors is warranted yet is difficult due to the challenges of detecting T1D in its initial stages.

### 6.3. Therapeutic Strategies Targeting Alpha Cells

One proposed strategy for targeting alpha cells in T1D is to restore their ability to respond to hypoglycemia by activating their endogenous paracrine and autocrine pathways [9]. One potential mechanism for this is through serotonin. The hypoglycemic effects of using selective serotonin receptor inhibitors (SSRIs) have been proposed to benefit T1D individuals; however, there is a risk of hypoglycemic shock as they may not be able to counter the drop in glucose [9]. Another proposed strategy is attempting to restore the glucose dependency of glucagon secretion. One potential mechanism for this is to utilize glutamate to activate glucagon secretion [73]. Current studies demonstrate that decreased glucose concentrations stimulate glutamate release through its ionotropic glutamate receptor, which is highly expressed in human alpha cells [73]. Furthermore, studies in a preclinical mouse model show that lowering glucose concentrations stimulates the release of glutamate while reducing glutamate secretion worsens hypoglycemia [73]. However, one key issue is determining how to develop therapeutic strategies that do not interfere with the effects of glucose on alpha cells. A different strategy targets alpha cells through the chemokine CXCL10, shown to be expressed by the alpha cells of T1D donors, which points towards an inflammatory response [69,74].

A further proposed therapeutic strategy utilizes the ability of alpha cells to trans-differentiate into beta cells [56,58,75]. As this already occurs in T1D, exploiting this may help restore more of the lost functions of beta cells [26]. Since alpha and beta cells arise from the same precursor, they may present the best alternative, especially over non-endocrine cells that traditionally have been the focus of generating new beta cells [26]. Many cell targets have been targeted to drive this conversion of alpha to beta cell differentiation, such as *Pax4*, *Arx*, and *Dnmt1*, but the shift has turned to *MafA* and *Pdx1* [26]. Moreover, the Pax4 transcription factor appears to play a role in alpha to beta cell trans-differentiation in the mouse model [47]. Furthermore, it has been demonstrated in streptozotocin-treated mice that one key component of the mechanism of partially reversing diabetes with incretin therapy (liraglutide, sitagliptin, dapagliflozin) includes trans-differentiation of alpha cells into beta cells [63]. However, the results have been inconsistent in reporting the impact on systemic hyperglucagonemia. 

Currently, there is evidence that γ-aminobutyric acid (GABA) may play a role in the trans-differentiation of alpha cells to beta cells in T1D and can be utilized as a therapeutic approach for T1D; however, this is still controversial [76,77,78,79]. One study testing GABA in T1D pediatric patients demonstrated no differences in glycemic control or diabetes antibody titers; however, the doses of GABA used were low and may not have been high enough to observe a difference with GABA treatment [80]. 

## 7. Therapeutic Interventions and Alpha Cell Modulation 

### 7.1. Current Therapies Influencing Alpha Cell Activity

Glucagon receptor antagonists (GCAs) have been reported as a potential therapy influencing alpha cell function. Results indicate improved glycemic control with treatment using GCAs in diabetic humans and rodents [58,81]. Several trials have aimed to find an optimal GCA; after initially focusing on non-peptide small molecules, some trials were discontinued due to severe side effects such as increased LDL-C, fatty liver disease, elevated blood pressure, weight gain, and alpha cell malignant transformation [56]. Newer GCA drugs, however, offer a more favorable human safety profile and are on the rise but still have some concerns such as non-alcoholic steatohepatitis [56]. Currently, monoclonal antibodies and antisense oligonucleotides targeting GCAs have demonstrated the best efficacy with markedly reduced side effects. In mouse models, there is evidence that treatment with glucagon receptor antibodies can reverse hyperglycemia through mechanisms such as alpha-to-beta cell trans-differentiation [82]. Their efficacy in human T2D patients is being tested in Phase III trials, but the hope is to also investigate the efficacy in T1D. 

With this focus on glucagon receptors, one study sought to target these receptors with a monoclonal antibody (mAb) or with γ-aminobutyric acid (GABA) alone or in combination to evaluate the effects in streptozotocin (STZ)-induced T1D mice [83]. The results indicated that treatment with either the glucagon receptor mAb or GABA alone mitigated hyperglycemia and increased insulin levels by regenerating beta cells [83,84]. However, when administered in conjunction, there were no synergistic effects, and the results were slightly antagonistic despite improving hyperglycemia. Most uniquely, the effect of either treatment led to alpha cell hyperplasia, which is a promising target to combat alpha cell dysfunction. Although the results indicate the combined therapy was not as effective, this could be a potential route to develop further therapeutic strategies. This may also have a significant impact on various other diseases that are related to impaired GABA function, such as depression, anxiety, and autism spectrum disorders [85,86,87,88]. Previous meta-analyses have found significant correlations between T1D and mental health disorders and neurodevelopmental disorders, such as depression, anxiety, and feeding or eating disorders [89,90]. Targeting GABA function in T1D may concurrently improve the prevalence of these associated diseases to improve the quality of life.

Multiple randomized, double-blinded studies have highlighted the favorable impact of dipeptidyl peptidase-4 (DPP-4) inhibitors on glucagon and insulin serum levels, particularly in the postprandial period [91,92]. Notably, research indicates a reduction in prandial glucagon secretion following two years of treatment with vildagliptin [56]. Similar findings were observed with sitagliptin when added to insulin in the treatment regimen for T1D. Moreover, long-term sitagliptin use in patients with slow-progressing T1D or latent autoimmune diabetes has been associated with enhanced glycemic control and preservation of beta cell mass [93]. These studies point toward the potential of further investigation into employing therapeutic strategies used for T2D in the treatment of T1D. 

Somatostatin plays a crucial role in inhibiting glucagon secretion in islet cells. This mechanism is also relevant in T1D, where somatostatin’s function involves a coupled depolarization process: upon depolarization, somatostatin is secreted, leading to hyperpolarization of alpha cells and subsequent inhibition of glucagon release [56]. This regulatory pathway helps maintain glucose homeostasis by modulating the secretion of glucagon, a hormone involved in raising blood sugar levels. The dysregulation of this process is implicated in both T1D and T2D in rats, highlighting the importance of understanding somatostatin’s role in pancreatic hormone secretion for developing effective diabetes treatments [94].

### 7.2. Emerging Therapies and Their Impact on Alpha Cells

There are multiple studies being conducted, many in vitro, in the search for emerging targets in T1D, specifically on alpha cells. Currently, there are studies pointing toward an elevation of IL-6 in T2D adult mice in relation to alpha cell mass expansion and hyperglucagonemia, and current research indicates this may also play a role in T1D [56,95]. In humans, various studies have demonstrated an elevation in IL-6 in T1D individuals, and the T cell response is mainly dependent on the surface levels of IL-6 receptors [96,97,98,99]. Despite this correlation, one study on the effects of tocilizumab (monoclonal antibody against IL-6) demonstrated no change in T cell phenotype between the treatment and placebo groups in newly diagnosed T1D patients [100]. Interestingly, liraglutide was able to reduce IL-6 levels in adult T1D patients with polyneuropathy in a randomized, double-blinded, controlled study; however, it did not improve established neuropathy [101]. This could be promising in early stages of T1D diagnosis to modulate the inflammatory state to prevent progression. Studying IL-6 in relation to alpha cell proliferation in T1D may be a potential therapy [102]; however, this still requires in-depth investigation. 

Additionally, a promising avenue for emerging therapies is generating new insulin-producing beta-like cells. In T1D, there is an increased number of glucagon-secreting cells, and this points to alpha cells, which also contain insulin and *Pdx1*, a beta cell transcription factor [103]. Generating the new beta-like cells involves reprogramming other native islet cell types within the adult pancreas through genetic modification [104,105]. This process entails the ectopic expression of a combination of three crucial pancreatic beta cell transcription factors: *Pdx1*, *Ngn3*, and *MafA* [103,104]. Studies have revealed that *MafA* enhances the ability of *Pdx1* to induce beta cell formation from alpha cells. Furthermore, the co-overexpression of these three genes has demonstrated the conversion of Sox9-positive liver cells into insulin-producing cells. This trans-differentiation holds significant potential for regenerative therapies targeting diabetes mellitus. It has been demonstrated that using the human glucagon (GCG) promoter to drive the transcription of factors *Pdx1* and *MafA* via an adeno-associated viral (AAV) 8 vector presents a promising approach for reprogramming alpha cells into insulin-producing beta-like cells. 

Another study demonstrated in mice that administering lithium in insulin-deficient hyperglycemia has effects on glucagon by reducing alpha cell mass without affecting insulin-secreting beta cell mass [106], while a further study found that eicosapentaenoic acid, a ω-3 polyunsaturated fatty acid, promotes trans-differentiation of alpha cells to beta cells and serves as a point of potential T1D intervention [107]. Moreover, the Pax4 transcription factor appears to play a role in alpha to beta cell trans-differentiation in the mouse model [47]. Further investigation into the trans-differentiation of alpha cells to beta cells may prove to be an effective therapeutic target.

As research has shown that the alpha cell population remains preserved in both early and late stages of autoimmune diabetes, targeting alpha cells during the early stages of T1D appears to reprogram the alpha cells to cells that produce insulin so that these cells proliferate and can potentially secondarily reduce beta cell dysfunction [75]. AAV vectors are utilized for delivering long-term expression of transgenes, accommodating sequences up to 4.5 kb in length. Therefore, a therapeutic strategy involves infecting pre-diabetic mice pancreatic alpha cells using the AAV8-GCG-PM viral delivery method to reprogram them into insulin-producing cells early in disease development. As the disease progresses, alpha cells proliferate, and the infected cells also undergo reprogramming, making the approach more effective over time. This process was effective in normalizing glucose levels in both chemically induced and autoimmune diabetic mice, indicating further research into utilizing the pathway of trans-differentiation as a potential therapeutic strategy.

Another potential therapeutic strategy that utilizes the pathway of alpha cell trans-differentiation is taurine, a semi-essential amino acid. Taurine has been shown to alleviate obesity and hyperglycemia in humans, but more investigation into whether it can be utilized as a therapy for diabetes has not been characterized. A further study exposed streptozotocin-treated mice to chronic taurine for ten days, which opposed the typical changes in islet morphology and beta:alpha cell ratio. Most importantly, taurine enhanced alpha cell trans-differentiation to beta cells, further confirmed by an increase in cytosolic calcium concentrations in the pancreatic alpha cells [108]. These results suggest that the trans-differentiation pathway may be a key therapeutic target in T1D, albeit there may be many different strategies [109].

Furthermore, recent in vivo studies have suggested that alpha cells upregulate GLP-1 gene expression in pathologic conditions so that they are transformed into beta cells to provide paracrine GLP-1 stimulation to remaining beta cells [56]. As noted earlier, GLP-1 has well-studied effects on insulin; GLP-1 receptors have been previously shown to also inhibit glucagon secretion on activation [47,56]. Previous studies utilizing exenatide and liraglutide have demonstrated GLP-1 receptor agonists’ promising role in treating T1D, with evidence of weight loss, lower frequency insulin use, and improvements in glycemic control; however, further investigation is needed to better evaluate the benefits and risks of utilizing GLP-1 receptor agonists [110]. Somewhat conversely, a study with 14 participants demonstrated that treating C-peptide-positive T1D with liraglutide did lower hemoglobin A1C, body weight, and insulin use but found no significant change in hypoglycemia [111]. Further studies utilizing a larger study cohort can provide more insight into how GLP-1 receptor agonists may aid in controlling T1D. Closely related, a weak response to serotonin in alpha cells leads to a loss of glucose-dependent inhibition of glucagon release, which leads to hyperglucagonemia in high glucose environments [56]. This is tightly related to acetylcholine release concurrently with glucagon and can potentially promote glucagon secretion systemically as acetylcholine promotes nitric oxide release [56]. 

### 7.3. Potential of Alpha Cell Regeneration and Replacement Therapy

Alpha cell regeneration and replacement therapies have been suggested to be a potential route of treatment in T1D. However, the best targets and methods are still being studied, and further investigation is especially warranted with additional evidence emerging about both alpha cell and glucagon secretion’s roles in T1D. Glucagon secretion is modulated by paracrine signaling from neighboring beta and delta cells, which is critical to inhibition in hyperglycemic environments (e.g., postprandial). Robertson and colleagues, utilizing an animal model, have shown that re-establishing this switch in T1D is critical to restore proper glucagon levels in T1D; however, the mechanism is still unknown [112]. This points towards the importance of further investigating alpha cells in T1D. Most notably, glucagon secretion seems to follow relative changes in glucose concentration and not absolute glucose levels. In addition, glucagon secretion is heavily dependent on the sodium and calcium channels of alpha cells, since when these channels are desensitized and unable to generate a full action potential, there is glucagon secretion dysfunction [9]. Characterizing this dysfunction fully could make huge strides in T1D treatment and change the scope of disease management.

## 8. Challenges and Opportunities

While research is being conducted on alpha cells, there remains much that is unknown. Although there is evidence and information regarding the transformation of beta cells in T1D into non-differentiated forms or other pancreatic endocrine cells (dedifferentiation and trans-differentiation, respectively), limitations exist due to the replicability of the pancreatic islet cell environment and the standardization of pancreatic cell lineage markers, creating high variability [56]. These alpha cells in T1D may be “bi-hormonal”, having the ability to secrete both insulin and glucagon, but results have been inconsistent, particularly in cases of hyperglucagonemia.

Studying alpha cells in T1D management has shown a variety of diverse results; however, one key limitation is how these therapeutic strategies interact with glycemia on alpha cells. Moreover, alpha cells provide an inhibitory input in hyperglycemic conditions while any potential therapeutic strategy would be utilized in both hyperglycemic and hypoglycemic conditions [9]. While dysregulated glucagon secretion by alpha cells plays a role in these conditions, it is also important to note that hypoglycemia in T1D occurs commonly after insulin administration. Further investigation into the alpha cell’s autocrine and paracrine regulation and electrical response in relation to the glycemic environment is crucial to developing further strategies.

The massive strides in research on alpha cells in T1D have demonstrated the strong potential of targeting them as a therapeutic strategy, which warrants further research [113]. While there are myriad results for targeting alpha cells in T2D, it is important to also look further into T1D; for example, somatostatin’s role in inhibiting glucagon secretion in T2D warrants further study on its potential role in T1D. Activation of glucagon receptors normally stimulates glucagon gene transcription through the cAMP response element-binding (CREB) pathway, activated by protein kinase A (PKA) [56]. However, studies have shown that blocking glucagon receptors can improve glucose homeostasis. Liu et al. have studied potential therapeutic strategies utilizing an alpha cell line created from an adenoma in transgenic mice [78]. This is because glucagon, which typically stimulates its own release by acting on glucagon receptors on alpha cells in an autocrine manner, is inhibited when those receptors are blocked. As a result, blocking glucagon receptors can help regulate glucagon secretion and thus contribute to better glucose control. This attests to the further potential of investigating these mechanisms in alpha cells for more targets.

Despite the increase in studies on alpha cells, islet cells from donors are still difficult to isolate and study in T1D. A current novel approach involves purifying human alpha cells from the islets of deceased donors and reaggregating them, either alone or in combination with beta cells [9]. This method aims to examine their response to decreasing glucose concentrations, a critical aspect of glucose counter-regulation given the importance of cell-to-cell contact [9]. Meanwhile, in parallel, a mouse model with beta cell destruction induced by streptozotocin is utilized to mimic the loss of the ability to respond to hypoglycemia [9]. This dual approach allows for a comprehensive investigation into the function and interactions of alpha and beta cells under conditions relevant to glucose regulation and hypoglycemia.

More importantly, investigating the various endocrine interactions involved in glucagon secretion and T1D is a top priority. Neural cell adhesion molecule (NCAM) has been implicated in the cell-to-cell interaction involved in regulating glucagon secretion, as NCAM-knockout mice have been shown to have defective glucagon secretion due to altered granule exocytosis [56]. As previously mentioned, GLP-1 may play an important role in T1D [114]. While GLP-1 and GLP-1 receptors have been well studied, especially their effects on insulin in T2D, they have been more difficult to characterize in T1D. GLP-1 analogues have been shown to have beneficial effects on HbA1C levels and glucagon serum levels in T1D, and their influence on both insulin and glucagon are thought to contribute equally to those effects. With the rise in the use of GLP-1 receptor agonists for other conditions as well, the full characterization of this target is most imperative.

Previously, it has been shown that activation of GLP-1 receptors by GLP-1 inhibits glucagon secretion due to direct activity on alpha cells. Although difficult to study, it has been demonstrated that there is a significant voltage change that inhibits P/Q-type calcium channels that block glucagon exocytosis [115]. However, GLP-1 is unable to block the effect of glucagon during hypoglycemia [56]. This significant advancement in the understanding of glucagon secretion in T1D requires further characterization, since, as previously noted, it is currently hypothesized that somatostatin mediates the ability of GLP-1 to inhibit glucagon secretion, yet the exact mechanism is unknown. It has been reported that the addition of 1.2 and 1.8 mg of liraglutide in T1D patients is associated with a reduction of postprandial plasma glucagon concentration, and similar results have been demonstrated using pramlintide [56]. Despite the benefits in hyperglycemic environments, further study into GLP-1 antagonists in hypoglycemic states is needed, as it has been shown that liraglutide for four weeks is unable to overcome the counter-regulatory effects of glucagon in this state. As hypoglycemia is a big clinical implication in T1D, it is important to develop therapeutic strategies that are effective during these glycemic states and also to prevent them from occurring.

Another related target is dipeptidyl-peptidase-4 (DPP-4). The DPP-4 enzyme normally breaks down endogenous GLP-1 and GIP, both incretin hormones that play a role in inhibiting glucagon release. Inhibiting DPP-4 has been demonstrated to prolong the half-life of GLP-1 and GIP to inhibit alpha cell activity and inhibit glucagon secretion, also increasing insulin postprandially [61]. DPP-4 inhibitors have been used in T2D and have good tolerability and can even reduce the risk of hypoglycemia. Interestingly, various studies have shown that DPP-4 inhibitors exhibit different effects depending on glucose levels: in hyperglycemia, DPP-4 inhibitors inhibit glucagon secretion, while in hypoglycemia, they enhance glucagon secretion [116,117]. However, the glucagonostatic effects of DPP-4 require further study for optimization in targeting dysfunctional alpha cells and for usage in T1D and potential long-term effects.

Another study demonstrated that activation of erythropoietin-producing human hepatocellular receptor type-A4 (EphA4), a tyrosine kinase, on alpha cells using doxazosin can reduce glucagon secretion and normalize blood glucose in human donors and mouse models [118]. They also demonstrated similar results utilizing a synthesized high-affinity molecule agonist of the EphA4 receptor, WCDD301, in diabetic, non-obese, and streptozotocin-treated mice. These findings target hyperglucagonemia, which is a key etiology of T1D and can potentially be used as a therapeutic strategy in T1D patients. 

## 9. Conclusions and Future Directions

There is emerging interest in deciphering the role of alpha cells in T1D pathophysiology. Research has revealed that alpha cells play a crucial role in glucose homeostasis and may serve as promising therapeutic targets in the management of T1D. A promising mechanism in mouse models has been activating endogenous pathways utilizing various strategies; however, this has not yet been tested in vitro or in humans. A more developed method to target alpha cells in T1D is alpha trans-differentiation. While there has been extensive investigation into this strategy, the results have been mixed in mice and human studies. Furthermore, T1D is an autoimmune disease characterized by the immune-mediated destruction of beta cells, where autoantibodies, complement proteins, and immune cells such as T cells play critical roles in targeting and destroying pancreatic beta cells [119,120]. This autoimmune process presents a significant challenge for regenerative therapies such as alpha-to-beta cell trans-differentiation. While trans-differentiation holds promise for replenishing lost beta cell mass, there is a risk that newly formed beta cells could be similarly targeted by the ongoing autoimmune attack, thus reducing the long-term efficacy of this approach [121]. Strategies to protect newly formed beta cells, such as immunomodulatory therapies, are being explored to address this issue, but they remain experimental at this stage. Additionally, employing immunocompromised or genetically modified mouse models may facilitate the study of the regenerative capacity of trans-differentiated cells without immediate autoimmune interference [122]. However, translating these findings to human T1D patients presents additional challenges, as it will be essential to account for the complexities of the immune response inherent to T1D. Addressing these challenges is crucial to optimizing trans-differentiation therapies for sustained glucose regulation in T1D. Glucagon receptor antagonists and monoclonal antibodies targeting this pathway have demonstrated efficacy in human trials and may have significant influence on alpha cell activity. However, many of these potential strategies targeting alpha cells have been studied predominantly in mouse models and only occasionally in vitro. Of note, DPP-4 inhibitors and GLP-1 agonists have been investigated in human clinical trials to determine if certain T2D treatments are effective in T1D, and the results are promising, but these investigations require larger study sizes and more in-depth evaluation. Furthermore, the widespread expression of GCRs in various key organs such as the liver may have adverse long-term consequences, which have not been heavily investigated yet. Indeed, significant questions must be addressed before these concepts can be translated to human interventions.

Despite advances in our understanding of alpha cell physiology and their role in disease progression, critical questions remain unresolved, such as the response of alpha cells to fluctuating glycemic conditions, the optimal therapeutic strategies, and the feasibility of translating findings from preclinical models to human patients. Furthermore, the dynamics of glucagon secretion from alpha cells, along with their autocrine and paracrine interactions within the pancreatic islet, represent emerging and contentious areas of research. Unraveling these complex signaling networks is essential for developing therapies that precisely modulate the pancreatic hormone milieu to sustain glucose homeostasis. Disruptions in this intricate balance play a significant role in diabetes pathophysiology, indicating that therapies aimed at restoring islet cell equilibrium may hold substantial therapeutic promise.

In summary, the outlook for advancing diabetes research and therapeutic approaches is exceptionally promising, with alpha cells emerging as a pivotal focus in the development of more effective treatments. By leveraging cutting-edge technologies and enhancing our understanding of alpha cell biology, especially in relation to GLP-1 and NCAM pathways, the scientific community is poised to uncover transformative interventions. These breakthroughs hold the potential not only to enhance diabetes management but also to introduce curative strategies, representing a significant leap forward in the fight against type 1 diabetes (T1D).

## Data Availability

All relevant data are within the paper.
